# Two cases of pituitary stalk interruption syndrome: importance of early detection and management

**DOI:** 10.1097/MS9.0000000000002123

**Published:** 2024-05-06

**Authors:** Mohammed Deeb Zakkor, Firas Khana, Mohammed Abdulrazzak, Sedra Kreid, Alae Kayyali, Hachem Al Hussein

**Affiliations:** a Department of Endocrinology, Aleppo University Hospital, Aleppo; b Department of Medical Imaging and Diagnostic Radiology, Aleppo University Hospital; c Faculty of Medicine, University of Aleppo, Aleppo, Syria

**Keywords:** case report, hormone replacement therapy, pituitary gland, pituitary stalk interruption syndrome

## Abstract

**Introduction and importance:**

Pituitary stalk interruption syndrome (PSIS) is a rare congenital condition affecting the pituitary gland and its stalk, leading to hormonal imbalances. PSIS can present with a wide range of symptoms, including delayed puberty and short stature.

**Case presentation:**

This paper discusses two cases of PSIS in patients with a history of growth hormone deficiency. The first case is of a 26-year-old male presenting with fatigue and loss of appetite, while the second case is of a 14-year-old male presenting with delayed puberty. Blood tests revealed hormonal imbalances, and a subsequent MRI confirmed the diagnosis of PSIS. Hormonal supplements were prescribed to manage the condition, and follow-up appointments were scheduled to monitor progress.

**Clinical discussion:**

PSIS can present with a wide range of symptoms, and can be diagnosed at different ages. Early diagnosis and management of PSIS are crucial to prevent long-term complications such as short stature, impaired cognitive function, and infertility. The use of hormonal supplements, as seen in both cases, is essential to manage the hormonal imbalances associated with PSIS. Testosterone replacement therapy is used to treat hypogonadism, while thyroxine and hydrocortisone are used to manage hypothyroidism and adrenal insufficiency, respectively.

**Conclusion:**

Early diagnosis and management of PSIS through hormonal supplements are crucial to prevent long-term complications. It is essential to monitor patients’ progress through follow-up appointments to ensure optimal management of the condition.

## Introduction and importance

Highlights

- Pituitary stalk interruption syndrome (PSIS) is characterized by the classic triad of anterior pituitary hypoplasia, ectopic posterior pituitary, and a thin or absent pituitary stalk.
- This paper discusses two cases of PSIS in patients with a history of growth hormone deficiency.
- MRI is a valuable tool for diagnosing PSIS.

Pituitary stalk interruption syndrome (PSIS) is a rare congenital defect of the pituitary gland that results in a deficiency of the anterior pituitary hormones. It can occur at different ages, manifesting with hormone deficiencies such as isolated growth hormone deficiency (GHD) by 100% or multiple anterior pituitary hormone deficiencies, hypogonadotropic hypogonadism, corticotrophin, and thyrotrophin, respectively, according to the incidence rate^1^.

The pituitary gland, located in the sella turcica at the base of the skull, comprises two lobes – the anterior and posterior lobes – which have distinct embryologic origins. The anterior lobe arises from the oropharynx, specifically Rathke’s pouch, and ascends during development to meet neural tissue growing downward, giving rise to the posterior lobe. Once fully developed, the posterior lobe houses nerve endings of neurons that originate in the hypothalamus. The pituitary stalk acts as a conduit between the hypothalamus and the pituitary gland, facilitating the transmission of axons from the hypothalamus to the posterior lobe and regulatory hormones from the hypothalamus to the anterior lobe through a portal system. The anterior lobe, constituting about two-thirds of the total pituitary volume, is larger than the posterior lobe^2^. PSIS is characterized by the classic triad of anterior pituitary hypoplasia, ectopic posterior pituitary, and a thin or absent pituitary stalk^3^.

The precise prevalence is unknown, but it is estimated to occur in 0.5 out of every 1 000 000 births^4^. Its prevalence as a cause of GH deficiency is estimated to be around 4%. The diagnosis could be estimated based on clinical findings; however, MRI scan can provide a definitive diagnosis^5^.

The importance of early diagnosis of pituitary hormones deficiencies come from two main resources. Firstly, if it is left untreated, it can result in significant mortality and morbidity. Secondly, inadequate height during the initial stages of puberty can lead to a shorter final height. Therefore, early identification and management of GHD are crucial to enable normal growth and development before puberty. So, as soon as this syndrome is diagnosed, the patient must immediately undergo hormone replacement therapy to avoid complications and the consequences of pituitary dysfunction^6^.

These cases demonstrate the diverse nature of PSIS with regards to the age of diagnosis, clinical manifestations, prognosis, and the treatment considerations that arise due to the rarity of this condition highlighting the diagnostic challenges associated with such cases. This work has been reported in line with the Surgical CAse REport (SCARE) 2023 criteria^7^.

## Cases presentation

Two cases of PSIS in patients with a history of GHD were shown in our endocrinology clinic.

### Case 1

A 26-year-old male patient presented to the endocrinology clinic with complaints of fatigue and a loss of appetite. The patient had a medical history of GHD that was diagnosed at the age of 5 years, for which he was treated with growth hormone (GH) for 7 years. At the age of 13, he experienced delayed puberty and sought medical help from an endocrinologist. Upon investigation, the patient was diagnosed with hypogonadotropic hypogonadism, adrenal insufficiency, and hypothyroidism. Hormonal supplements were prescribed, including testosterone 75 mg IM injection every 15 days, thyroxine 100 μcg daily, and hydrocortisone 20 mg daily, divided into two doses of 15 mg in the morning and 5 mg in the evening.

Upon examination, the patient’s blood pressure was 90/60 mmHg. Blood tests revealed low sodium levels of 133 mEq/l, potassium levels of 4.5 mEq/l, and elevated prolactin levels of 31 ng/ml. Additionally, free thyroxine (FT4) levels were found to be 1.2 ng/dl (normal range: 0.8–1.8 ng/dl), follicle-stimulating hormone (FSH) levels were at 0.3 μIU/ml (normal range: 1–7 IU/l), and luteinizing hormone (LH) levels were at 0.1 μIU/ml (normal range: 1.8–8 IU/L). Adrenocorticotropic hormone (ACTH) for an early morning sample (8 a.m.) was found to be at 16 pg/ml (normal range: 10–60 pg/ml). A morning baseline cortisol level test revealed a low value of 2.3 μg/dl (normal range: 5–25 μg/dl) (Table 1). The secondary sexual characteristics of the patient were consistent with Tanner Stage V.

**Table 1:** Patient’s laboratory tests revealing the typical pituitary stalk interruption syndrome’s findings.

	Patient 1	Patient 2	Reference range
Free testosterone	0.06	0.03	6.8–21 pg/ml
FT4	1.2	0.4	0.8–1.8 ng/dl
TSH	0.8	1	0.4–4 ml/l
ACTH	16	12	10–60 pg/ml
Cortisol (8 a.m.)	2.3	1	5–25 μg/dl
Prolactin	31	26	Less than 20 ng/ml
Na	133	131	135–145 mEq/l
K	4.5	.3.8	3.5–5.3 mmol/l
FSH	0.3	0.8	1–7 IU/l
LH	0.1	0.2	1.8–8 IU/l
GH after clonidine stimulation test		3	More than 3 ng/ml

**Figure 1:**
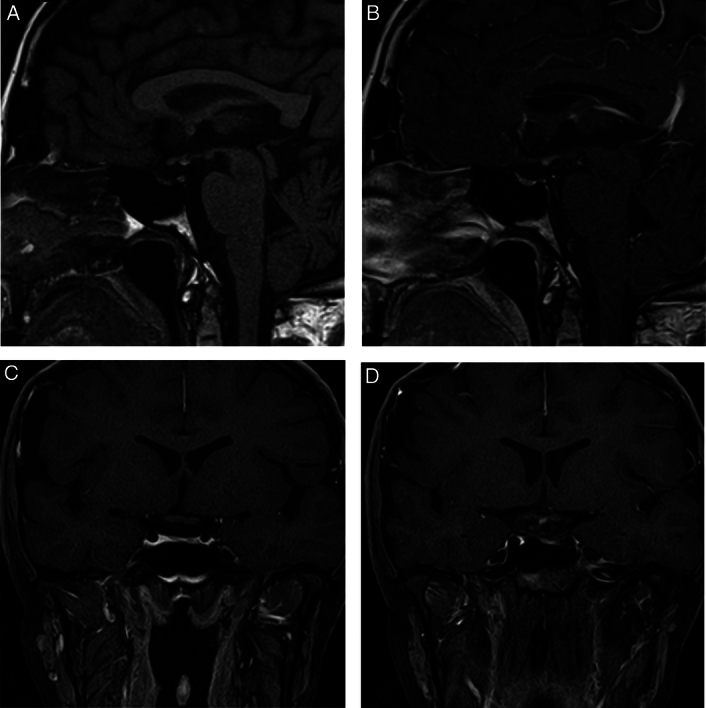
MRI findings: (A,B) Precontrast and postcontrast. T1-weighted sagittal images showing the presence and enhancing nodule at the level of the hypothalamus suggestive of an ectopic posterior pituitary with a hypoplastic anterior pituitary measuring only at 2.1 mm in height. (C,D) Precontrast and postcontrast fat-suppressed coronal images showcasing the absence of the pituitary stalk, and confirming the presence of the ectopic posterior pituitary.

Subsequent MRI of the pituitary gland revealed characteristic findings of PSIS, which included a thin or interrupted pituitary stalk, hypoplasia of the anterior pituitary, and absence of the posterior pituitary. On ultrasound, the gallbladder demonstrates echoes and posterior dense shadowing, with poor delineation of the gallbladder wall itself that suggest porcelain gallbladder (Fig. 1).

As a result, the dosage of hydrocortisone was adjusted accordingly, leading to a gradual improvement in the patient’s condition.

### Case 2

A 14-year-old male presented to the clinic with a complaint of delayed puberty. The patient had a medical history of receiving GH therapy for 3 years. Upon examination, the patient was found to have extremely short stature, with a height of more than two SD points below the expected range. Additionally, the patient had a distorted body proportion of less than 3% and an impaired IQ. The absence of secondary sexual characteristics was also noted. An radiograph of the hand revealed a delayed bone age of 3.5 years.

Laboratory assessment revealed panhypopituitarism, with the clonidine stimulation test for GH inducing GH levels of 3 mU/I. Furthermore, FSH and LH levels were found to be 0.8 uIU/ml and 0.2 μIU/ml, respectively. FT4 levels were at 0.4 ng/dl, ACTH levels at 12 pg/ml, and morning cortisol levels were low at 1 μg/dl. Additionally, testosterone levels were found to be 0.03 ng/ml, and prolactin levels were elevated at 26 ng/ml (Table 1).

**Figure 2:**
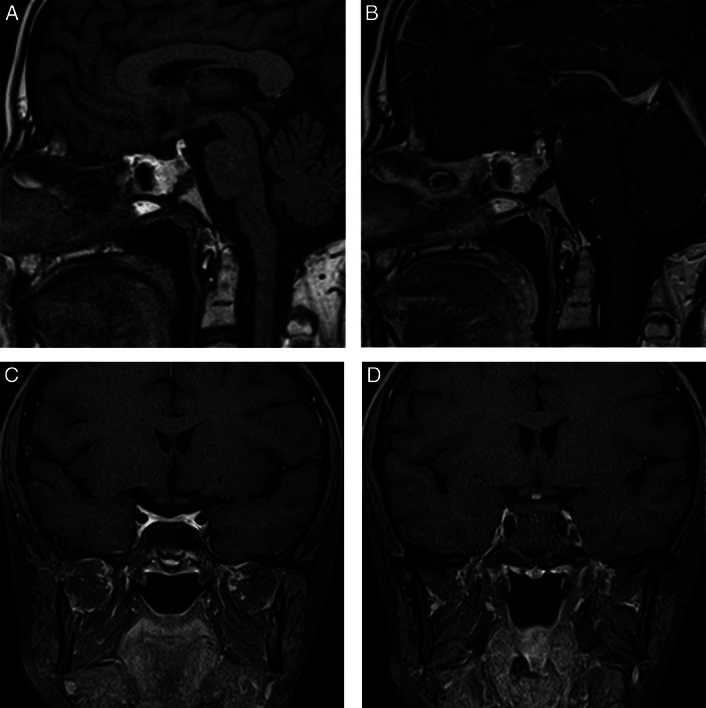
MRI findings: (A,B) Precontrast and postcontrast. T1-weighted sagittal images showing the presence and enhancing nodule at the level of the hypothalamus suggestive of an ectopic posterior pituitary with a hypoplastic anterior pituitary measuring only at 2.5 mm in height. (C,D) Precontrast and postcontrast fat-suppressed coronal images showcasing the absence of the pituitary stalk, and confirming the presence of the ectopic posterior pituitary.

Subsequent MRI revealed the triad of a thin or interrupted pituitary stalk, absent posterior lobe, and hypoplastic anterior lobe, confirming the diagnosis of PSIS (Fig. 2).

The patient was started on hormone replacement therapy with testosterone 75 mg IM injection every month until he becomes 18 years old, thyroxine 75 μcg daily, and hydrocortisone 25 mg daily, divided into two doses of 15 mg in the morning and 10 mg in the evening. The patient’s progress is being monitored through follow-up appointments.

## Discussion

PSIS is a rare congenital abnormality of the pituitary that was first reported by Fujisawa *et al*. in 1987^8^. PSIS is characterized by the classic triad of anterior pituitary hypoplasia, ectopic posterior pituitary, and a thin or absent pituitary stalk. The condition presents with a range of clinical phenotypes and hormone deficiencies, which vary in severity and onset timing^3^.

MRI is a valuable tool for diagnosing PSIS. The characteristic MRI features of PSIS include underdevelopment or absence of the anterior pituitary, lack of the hyperintense posterior lobe within the sella turcica, and its presence at the median eminence or pituitary stalk level as a hyperintense nodule, as well as a thinned or absent pituitary stalk. However, there are variations in the MRI presentations of PSIS, such as the height of the anterior pituitary (ranging from absent to normal), the appearance of the posterior pituitary lobe (ectopic at the base of the hypothalamus or along the pituitary stalk, absent, or normal), and the form of the pituitary stalk (interrupted, thin, absent, or normal). PSIS can also manifest as an isolated ectopic posterior pituitary^9^.

The age of diagnosis highly varies across the literature and depends on the severity of hormone deficiency. However, the clinical presentation of PSIS is diverse and complex due to the limited number of reported cases^10^. PSIS presenting at birth is often characterized by hypoglycemia and failure to thrive, while growth retardation is typically observed in childhood and delayed puberty is common in adolescence and early adulthood. Despite presenting with multiple hormone deficiencies, the diagnosis for the patients in this study was established later in life. Guo *et al*. reported a median age of 19.7 years for diagnosis, which is consistent with our patient. The delayed age of diagnosis for PSIS in various studies highlights the need for increased awareness and timely diagnosis in clinical practice^11^.

The etiology of PSIS still remains unclear. Individuals with congenital pituitary hormone deficiency and PSIS are more likely to have been born via breech delivery or cesarean section and to have experienced neonatal hypoxemia compared to the general population. Breech delivery can cause head deformation, which may lead to injury of the pituitary stalk. Likewise, hypoxemia resulting from anoxia following birth may also cause injury to the pituitary stalk and pituitary gland. Some researchers suggest that PSIS is a secondary condition resulting from these events leading to injury in this region during birth^12^. However, the presence of micropenis and cryptorchidism in association with PSIS, as well as familial or syndromal forms, suggests that the pathogenesis of PSIS has an antenatal origin. Therefore, abnormal birth data are a consequence rather than a cause of PSIS^6^.

In fewer than 5% of cases, genetic abnormalities were detected, including mutations, deletions, or sequence variations in genes such as HESX1, OTX2, LHX4, LHX3, PROKR2, GPR161, CDON, TGIF, GLI2, FGFR1, ARNT2, CHD7, as well as chromosomal defects such as 18p deletion, X chromosome translocations affecting SOX3, and 17q21.31 microdeletion^13^. However, genetic testing was not performed in our cases due to the lack of resources resulting from war conditions. In our cases, we were unable to conduct genetic testing on patients with PSIS due to limited resources. Despite this limitation, the study provided a detailed description of the clinical, endocrine, and imaging characteristics of the patients. Patients did not exhibit associated malformations, and additional abnormalities on head MRI scans besides PSIS.

A complete description of the clinical, endocrine, and imagistic phenotype is useful in the attempt to identify the underlying genetic defect in PSIS patients, despite extreme phenotypic variability, which should always be kept in mind. In the two patients described, sense of smell and vision were normal, they had no diabetes insipidus, no associated malformations and no other head MRI abnormalities beside PSIS. Nevertheless, there is an absence of extrapituitary malformation^14^.

PSIS is associated with a high prevalence of extrapituitary manifestations. The central nervous system and ocular structures are primarily affected by midline defects. Additionally, extracerebral malformations, such as those affecting the heart, skin, and extremities, are also observed. Therefore, it is crucial to conduct regular screenings of the cardiac, ophthalmologic, and cerebral systems^15^. The first reported case had porcelain gallbladder in young age. To the best of our knowledge, this association of PSIS has never been reported in the literature.

Upon confirmation of PSIS diagnosis through MRI, patients must undergo meticulous monitoring for potential pituitary deficiencies, particularly ACTH deficiency, which may pose a risk to life. Gonadotropin deficiency can be detected in male infants within the first 6 months of life if they exhibit micropenis and cryptorchidism, and within the first 2 years in PSIS females. In the absence of these symptoms, further investigations should be deferred until after the age of 12. Notably, PSIS may advance from solitary GHD to multiple pituitary hormone deficiencies even during the second or third decade of life^3^.

## Conclusion

These two cases reflect the diverse characteristics of PSIS, including the age of diagnosis, clinical manifestation, outcomes, and management concerns associated with this rare anomaly. MRI is the optimal method for evaluating structural alterations in hypothalamo-pituitary morphology and related extrapituitary malformations. In addition, consistent monitoring of other hormonal deficiencies is crucial for patients who present with isolated GHD.

## Ethical approval

This study is a two case reports, and our institution does not require ethical approval for such research, but they require obtaining the consent of the patient and the doctor supervising the case.

## Consent

Informed consent: Written informed consent was obtained from the patients and patient’s families for reporting this case and its associated images. A copy of the written consent is available for review by the Editor-in-Chief of this journal on request.

Consent for publication: All authors provide consent for publication.

## Sources of funding

There are no funding sources.

## Author contribution

All authors fulfill the authorship criteria because of their substantial contributions to the conception, design, analysis, and interpretation of the data. All authors contributed in the work’s conception and design, paper writing, and article revision, and final revision and approval.

## Conflicts of interest disclosure

The authors declare that they have no competing interests.

## Research registration unique identifying number (UIN)

Our research study does not involve human subjects.

## Guarantor

Mohammed Deeb Zakkor.

## Data availability statement

Not available.

## Provenance and peer review

Not commissioned, externally peer-reviewed.

## References

[1] Vergier J Castinetti F Saveanu A . Pituitary stalk interruption syndrome: etiology and clinical manifestations. 2019. 10.1530/EJE-19-0168 31480013

[2] Hong GK Payne SC Jane JAJ Jr . Anatomy, physiology, and laboratory evaluation of the pituitary gland. Otolaryngol Clin North Am 2016;49:21–32. 26614827 10.1016/j.otc.2015.09.002

[3] Voutetakis A Sertedaki A Dacou-voutetakis C . Pituitary stalk interruption syndrome: cause, clinical manifestations, diagnosis, and management. 2016. 10.1097/MOP.0000000000000378 27386973

[4] Mbbs AN Mbbs MA Mbbs JS . Pituitary stalk interruption syndrome presenting in a euthyroid adult with short stature Case report. Radiol Case Rep 2018;13:503–506. 29904499 10.1016/j.radcr.2017.12.002 PMC5999867

[5] Vergier J Castinetti F Saveanu A . Diagnosis of endocrine disease: pituitary stalk interruption syndrome: etiology and clinical manifestations. Eur J Endocrinol 2019;181:R199–R209. 31480013 10.1530/EJE-19-0168

[6] Ram N Ali SA Hussain SZ . Pituitary stalk interruption syndrome presenting as short stature: a case report. J Med Case Rep 2014;8:445. 25524465 10.1186/1752-1947-8-445 PMC4300583

[7] Sohrabi C Mathew G Maria N . The SCARE 2023 guideline: updating consensus Surgical CAse REport (SCARE) guidelines. Int J Surg 2023;109:1136–1140. 37013953 10.1097/JS9.0000000000000373 PMC10389401

[8] Fujisawa I Kikuchi K Nishimura K . Transection of the pituitary stalk: development of an ectopic posterior lobe assessed with MR imaging. Radiology.1987;165:487–9. 3659371 10.1148/radiology.165.2.3659371

[9] Pinto G Netchine I Sobrier ML . Pituitary stalk interruption syndrome: a clinical-biological-genetic assessment of its pathogenesis. J Clin Endocrinol Metab 1997;82:3450–3454. 9329385 10.1210/jcem.82.10.4295

[10] Gosi SK Kanduri S Garla VV . Pituitary stalk interruption syndrome. BMJ Case Rep 2019;12:e230133. 10.1136/bcr-2019-230133 PMC6506087 30988112

[11] Guo Q Yang Y Mu Y . Pituitary stalk interruption syndrome in Chinese people. clinical characteristic analysis of 55 cases 2013;8:e53579. 10.1371/journal.pone.0053579 PMC3544917 23341953

[12] Maghnie M Larizza D Triulzi F . Hypopituitarism and stalk agenesis: a congenital syndrome worsened by breech delivery? Horm Res 1991;35:104–108. 1806462 10.1159/000181883

[13] Castinetti F Reynaud R Saveanu A . Mechanisms in endocrinology: an update in the genetic aetiologies of combined pituitary hormone deficiency. Eur J Endocrinol 2016;174:R239–R247. 26733480 10.1530/EJE-15-1095

[14] Kelberman D Rizzoti K Lovell-Badge R . Genetic regulation of pituitary gland development in human and mouse. Endocr Rev 2009;30:790–829. 19837867 10.1210/er.2009-0008 PMC2806371

[15] Lichiardopol C Albulescu DM Pituitary stalk interruption syndrome: report of two cases and literature review. Acta Endocrinol (Buchar) 2017;13:96–105. 31149155 10.4183/aeb.2017.96 PMC6525749

